# Lessons learned from housing first, rapid rehousing trials with youth experiencing homelessness

**DOI:** 10.1186/s13722-023-00413-x

**Published:** 2023-09-30

**Authors:** Natasha Slesnick, Brittany Brakenhoff, Alicia Bunger, Laura Chavez, Caleb Cuthbertson, Ruri Famelia, Xin Feng, Maggie Fitzpatrick, Jodi Ford, Irene Hatsu, Eugene Holowacz, Soren Jaderlund, Kelly Kelleher, Ellison Luthy, Allen Mallory, Jared Martin, Alexis Pizzulo, Steven Stone-Sabali, Tansel Yilmazer, Qiong Wu, Jing Zhang

**Affiliations:** 1 Department of Human Sciences, College of Education and Human Ecology, The Ohio State University, 1787 Neil Ave, Columbus, OH 43210 USA; 2 College of Social Work, The Ohio State University, 1947 N. College Road, Columbus, OH 43210 USA; 3 Center for Child Health Equity and Outcomes Research, Nationwide Children’s Hospital, 700 Children’s Drive, Columbus, OH 43205 USA; 4 College of Nursing, The Ohio State University, 1585 Neil Avenue, Columbus, OH 43210 USA; 5 Department of Family and Child Sciences, Florida State University, Tallahassee, FL USA; 6 Department of Human Development and Family Studies, Kent State University, Kent, OH USA

**Keywords:** Housing first, Youth homelessness, Lessons learned, Interventions, Rapid re-housing

## Abstract

**Background:**

Youth, 18 to 24 years, experiencing homelessness (YEH) are recognized as having developmental challenges dissimilar to older adults. Yet, research on efforts to end homelessness and prevent or intervene in drug use and mental health problems among youth have lagged behind that of adults. The Housing First (HF) Model which underlies Permanent Supportive Housing (PSH) and Rapid Re-Housing (RRH) has become preferred over treatment-first models.

**Methods and results:**

We provide an overview of PSH and RRH studies to date and summarize our current understanding of their utility for use with YEH. Finally, we review our team’s current and past randomized trials testing RRH with YEH, providing lessons learned and recommendations.

**Conclusion:**

Current research efforts to guide best practices are hampered by a lack of fidelity to HF principles, lack of randomized design, and lack of focus on youth. Lessons learned and recommendations from our work are offered to facilitate the future work of those who seek to end homelessness and address drug use and mental health problems among youth.

## Background

Homelessness remains a major problem in the U.S. Youth homelessness is rising with 4.3% of 13–17 year-olds and 9.7% of 18–25 year-olds reporting homelessness in the prior year [1]. Homelessness co-occurs with substance use, poor physical and mental health outcomes, social exclusion, high rates of victimization and suicide, and premature mortality [2–5]. High rates of substance use, between 70 and 95%, are consistently reported among youth experiencing homelessness (YEH) [6], and 60–71% of YEH meet diagnostic criteria for a substance use disorder [7–10]. YEH carry a disproportionate burden in the opioid epidemic with rates of opioid use reported up to 79% [11, 12]. Overall, substance use is considered the norm rather than the exception among YEH. Similarly, rates of mental illness are three times higher among YEH compared to their housed counterparts, with 66–89% of the population having a mental health disorder, especially depression and anxiety [13–15].

Unfortunately, efforts to address homelessness and its associated problems including substance use and mental health problems among youth lag behind those of adults [16, 17] leading to missed opportunities for prevention efforts. One of the reasons that efforts to address youth homelessness lag behind adult efforts is that youth tend to avoid services that may be available to them, citing predation by older adults experiencing homelessness, lack of insurance, transportation barriers, limited knowledge of how to access available services, stigma, and fear of betrayal or return to foster care or home settings [18]. In addition, YEH have unique challenges associated with typical developmental milestones of this age period including identity development, negotiating romantic attachments, and the development of autonomy [18]. Because youth are so often disconnected from traditional health and behavioral health services, alternative settings for delivering substance use and mental health treatment and prevention are needed such as through shelters, drop-in centers, and streetside.

The first research trial testing a formal health intervention for YEH was a group-based HIV prevention conducted in a runaway shelter in 1994 [19]. In the years that followed, several other trials tested family, individual and group interventions addressing substance use, mental health and HIV risk for youth recruited from runaway shelters, the streets and drop-in centers [3, 20–23]. Intervention while youth are in the midst of their homeless crisis is essential for mitigating substance use, physical disorders, and mental health problems. However, providers assert that exiting homelessness confers greater protection for health and well-being by preventing long-term effects of homelessness from the compounding physical and mental stressors associated with surviving on the streets. Bundling behavioral health and health services within housing models has the potential to improve outcomes for youth, but there are gaps in our understanding about the most effective ways to design and implement these approaches, especially given developmental needs of adolescents and emerging young adults. This paper addresses that gap by documenting lessons learned and providing recommendations on the practical experiences of our study team in a randomized clinical trial testing supportive housing as an opioid/other substance use prevention for YEH, 18–25 years old [24, 25].

First, below, we provide a summary and discussion based upon a literature review of other efforts to house YEH, as well as adults. A search of the databases that included Academic Search Premier, Psych Info, Medline, Eric, and Social Work Abstracts was conducted with combinations of the following keywords: housing first, housing first philosophy, rapid rehousing, permanent supportive housing, youth homelessness, and adult homelessness. Studies were included in the review below if a housing intervention was tested for anyone experiencing homelessness, regardless of the targeted outcomes or other inclusion criteria. Unpublished master’s theses, dissertations and articles were not included. The requirement for a randomized design was not used because to date, few such studies have been completed.

### Housing: Risk prevention for youth

Drug prevention research has advanced considerably over the past 20 years. Improvements in decreasing availability of drug supply, bolstering family and school education around drug use among young people, and interventions in home and school settings all have shown effectiveness in preventing progression of youth drug abuse [26–28]. In part, these advances in preventive services delivered in home and school settings reflect the importance of social and environmental influences on drug use by youths. It is exactly these same influences which challenge preventive interventions for YEH. Successful interventions for families are not relevant when a young person is separated from family for safety, emotional, financial or other reasons and is unwilling or unable to return. Similarly, school interventions are not relevant for those living on the streets for the most part. And, while not all youth living on the streets struggle with substance use or mental health problems, the longer duration of time on the streets increases risk for the progression of drug use, violence, trauma, trafficking, and greater interaction with the criminal legal system [29]. Removing youth from the streets is broadly considered an important prevention tool for a range of negative health and well-being outcomes [30]. In fact, some have concluded that housing should be included as an important tool in the positive prevention arsenal, and “housing as healthcare” holds great promise [31]. Although the few existing studies indicate that substance use and mental health does not worsen among youth with housing [25, 32, 33], additional research is needed to identify the type of supports youth, as separate from adults, need in order to show optimal improvement. For example, some suggest that more structured housing and treatment intervention models may be necessary for youth as compared to adults and they may not align with a housing first philosophy [30]. A review of HF for adults and youth is offered in order to contextualize the discussion of lessons learned and recommendations derived from completed and ongoing HF trials with youth [24, 25, 33].

## Housing first (HF) philosophy

Housing programs that utilize a HF philosophy have no prerequisites for sobriety or participation in psychiatric treatment and eschew the treatment first philosophy, that one must first address substance use and mental health problems prior to receiving housing, that had been embraced by communities [34, 35]. The HF philosophy asserts that housing is a fundamental human right, recovery from mental illness is possible, and that consumers can make competent choices [34]. The central tenet of HF philosophy is that consumers have choice/control over “where they live, how they live, who they allow to live with them, who enters the home, to abstain from substance use or not, to comply with treatment demands or not, and the support that they receive” [36, p. 225]. Although Tsemberis [37] identified that 67% of cities’ plans to end homelessness utilized a HF first philosophy, limited research supports the implementation of HF programs given less than optimal fidelity to the HF philosophy.

HF principles underlie various housing approaches including permanent supportive housing (PSH) and rapid re-housing (RRH) [38, 39]. PSH is reserved for the most severely affected individuals with the highest need. Pathways to Housing implemented PSH using a HF philosophy to provide immediate access to independent apartments and supportive services for the most vulnerable homeless people with severe mental illness and substance use disorders. In PSH, the rental support, social services, and related case management are intended to be permanent as long as the individual continues to meet program requirements each year. RRH is for those with more resources and usually includes temporary rental support and social services with aligned advocacy or case management.

## Permanent supportive housing for adults

A recent review identified only four randomized controlled trials (RCTs) testing PSH using a HF philosophy for adults [16]. The authors concluded that studies to date indicate that HF approaches improve housing stability and do not appear to cause an increase in substance misuse or mental health problems with some studies showing small improvements in those outcomes. Other reviews included non-randomized studies resulting in 31 [40] and 34 studies [41]. Several trials show implementation drifts from the core tenets of HF [42–44] including choice over housing and the use of supportive services and abstinent based housing, thus muddling conclusions regarding the effectiveness of HF programming that is supposedly based upon a HF philosophy. Also, extant trials include various populations and methodologies, making conclusions regarding its effectiveness for specific populations difficult without further controlled trials.

Overall, published reviews on PSH converge on the conclusion that it is successful at increasing housing stability and reducing use of emergency health services. But the reviews also conclude that while substance use and mental health do not appear to deteriorate as a function of housing, they do not show differential improvement compared to services as usual. Some have suggested that this may be due to active interventions used as the comparison conditions, or the time frame for determining positive health and well-being outcomes may have been too short (usually 1 to 2 years). All reviews conclude that much more research is necessary to increase confidence about any conclusions regarding PSH’s impact on health and well-being beyond housing alone, and its impact under varying levels of fidelity to principles of HF.

Recently, Jacob et al. [45] concluded that PSH programs are economically beneficial from a societal perspective, even though the cost of PSH is often cited as a potential barrier for adoption. As noted by Ly and Latimer [41] the average annual costs for one adult experiencing homelessness are high, with available estimates in Canada ranging from $30,000–134,642 [46, 47], and in the US estimated to be $35,000 [48]. While some funders might consider spending on programs such as PSH justified only if the program pays for itself by offsetting other costs (shelter, emergency services, hospitalizations, incarcerations, etc.) evidence indicates that this might occur only for those with the most severe need [41, 49]. However, Ly and Latimer [41] argue that few healthcare innovations pay for themselves (e.g., cancer drugs usually do not meet traditional criteria for cost-offset). Furthermore, benefits to extending lives is considered sufficient merit to justify housing costs especially since PSH represents a more efficient allocation of resources than traditional services (shelters and emergency services) even for those with less severe needs.

## Rapid re-housing for adults

According to HUD, Rapid Re-housing (RRH), can also be informed by a HF philosophy [39]. Benchmarks of success as identified by the National Alliance to End Homelessness include rapid exits from homelessness to permanent housing and not returning to homelessness within a year. RRH was implemented across the country through the Homelessness Prevention and Rapid Re-housing Program (HPRP), as part of the American Reinvestment and Recovery Act (ARRA) of 2009 and was shown to be a cost-effective way to end homelessness for a range of households [39]. Unlike PSH, RRH offers temporary financial assistance to cover move-in costs, deposits, and rental and/or utility fees—typically for 3 months—but can be renewable for up to 18 months as necessary to allow individuals and families to maintain their permanent housing. As noted by HUD [38], the premise of the idea is “that resources are limited, and households should receive “just enough” assistance to successfully exit homelessness and avoid returning to the streets. Longer-term and more costly programs like PSH should be reserved for those individuals and families who need that level of assistance to exit homelessness and remain housed” [38, p. 1–2].

Few experimental studies have tested the effectiveness of RRH for various populations, as it receives much less attention than PSH even though its popularity has increased [50]. One experimental design [51] found that those receiving RRH were less likely than participants in the control group to enter emergency shelter at 27 months follow up, similar to the findings of PSH trials. A recent review of RRH [52] identified six studies—two RCTs [39, 53], and four quasi-experimental studies. Byrne et al. [52] concluded that the studies to date show little evidence that RRH produces better outcomes than transitional housing (temporary, supportive housing), but that PSH results in better housing outcomes than RRH showing lower rates of homelessness, being doubled up, and stays in emergency shelter at 20- and 37-month follow-ups. In conclusion, although still an open question, they did not find evidence that the different types of households studied—families and single adults living with HIV, do better with RRH than other housing interventions such as PSH—although housing, overall, improved compared to services as usual (SAU). Byrne et al. [52] called for research to more rigorously determine if RRH works better for certain households not examined in current studies. As reviewed below, RCTs testing housing interventions for YEH are essentially non-existent, even though many communities are implementing RRH for YEH, and HUD recommends RRH for youth [20, 39].

## PSH and RRH with youth: current evidence and ongoing studies

Only four quantitative studies report youths’ response to housing efforts [25, 32, 33, 54]. Highlighting the importance of adapting housing interventions for youth specifically, one study completed a subgroup analysis of youth, 18–24 years, participating in an RCT of PSH for adults in Canada [32]. Participants in the trial were of high and moderate need, with mental illness. Several characteristics of youth differed from older adults such that more youth had not finished high school (76% of youth vs. 54% of older adults), had a drug disorder (66% vs. 52%), and had been assaulted in the prior 6 months (44% vs. 36%). Also, a high number of youths, 61%, visited an ER in prior 6 months, indicating a lack of access to preventive health services. Relatedly, Gilmer [43] found that service costs increased for youth in PSH for transition age youth in California, indicating that once youth are stabilized in housing, they finally receive access to mental health and other services or entitlements not received while homeless. There were no adaptations of the PSH programmatic implementation for youth, and they found that even though PSH improved housing stability for youth, there were no significant improvements in other outcomes, including mental health and substance use, compared to usual care. In fact, youth showed worse employment outcomes, perhaps due to disincentives to work. They conclude that adaptations to address the unique needs of emerging adults is needed in future trials. Indeed, several researchers note that PSH and RRH interventions have not been adapted to the unique needs of youth which is essential given the unique challenges that youth face [18, 43, 54–56].

As Pathways to Housing’s PSH was originally developed for adults with severe mental illness experiencing chronic homelessness [34], RRH may be better suited for youth who have not yet progressed to that level of need. Long-term studies are needed to show whether RRH has important preventive effects on the youths’ transition to chronic adult homelessness, including severe mental illness and substance use, given their special developmental needs. Youth lose critical social support relationships during homelessness with relatives, teachers, foster families and others who may have been instrumental in their lives. These youth are expected to be financially and emotionally independent during their transition to adulthood which is made more difficult by homelessness and lack of family support [20]. YEH experience a stressed transition to adulthood “complicated by personal, social, and systemic factors that impede their gradual entry into self-sufficiency and healthy interdependence and rarely allowing for a period of “emerging adulthood” [18, p. 432]. Often the developmental tasks for YEH during this period are derailed by displacement from home into foster care or the juvenile justice system and ultimately to the streets where the focus becomes survival as well as the experience of traumatic events prior to and while living on the streets.

Furthermore, Gaetz, Ward and Kimura call for a broader consideration of outcomes for youth than observed among adults, which tend to focus on housing stability, physical outcomes, and mental health/substance use outcomes [56]. Instead, Gaetz and colleagues recommend a shift to including a focus on well-being and inclusion outcomes that supports stabilization, and that clarity on desired outcomes should then drive service provision (p. 76). In particular, these authors recommend the following targeted outcomes for housing trials with YEH: (1) housing stability, (2) health and well-being, (3) access to income and education, (4) complementary supports, and (5) improved social inclusion. Indeed, these outcomes will be assessed by “A Way Home Canada” and “Making the Shift – Youth Homelessness Social Innovation Lab” who, working together, are implementing three demonstration projects at 12 sites across 10 cities in Ontario and Alberta Canada, testing Housing First for Youth (HF4Y) [57, 58], an adaptation of the original Pathways program to meet the needs of developing adolescents and young adults [34]. HF4Y will be compared to (a) Enhancing Family and Natural Supports, and (b) Youth Reconnect, a school-based early intervention program with an expected sample of 1300 young people assessed over 48 months.

Exploring housing loss among YEH, Youngbloom and colleagues provide descriptive information from a non-experimental implementation of scattered site RRH in Austin, Texas [59]. Youth (N = 60) were offered up to 36 months of rental assistance and provided wraparound services. They found that 40% of youth were asked to leave or evicted within that 36-month period, and housing loss was predicted by youths’ depression, identifying as a sexual or gender minority, and foster care history. The authors did not include an examination of youth who left voluntarily from their housing, suggesting that housing instability may be greater than 40%, even before rental supports were removed. Details regarding implementation of HF principles in terms of housing (e.g., rules such as abstinent-based housing, prerequisites for housing, lease-signing) and provision of supportive services (voluntary or required) were limited, so it is not clear how well the HF philosophy was followed, which could explain the limited success of housing outcomes.

Two studies report outcomes of RRH using a HF philosophy for parenting YEH, between 18 and 24 years [25], as well as non-parenting YEH [24, 33]. The first study provided time-limited rental and utilities support (3 months), as well as fees for the rental application, damage deposit and furniture [25]. A HF philosophy was followed in that (1) housing was non-temporary as youth selected from among fair market housing options and were expected to remain in the apartment once rental assistance ends, (2) the youth had choice in the housing that they selected, (3) there were no prerequisites for receiving housing (employment, etc.) and (4) housing was not abstinence-based, and there were no requirements to receive counseling. Supportive services included a service linkage intervention, Strengths-based Outreach and Advocacy and substance use/mental health counseling (Community Reinforcement Approach) offered for 6 months. The housing and supportive services intervention (*n* = 80) was compared to housing only without supportive services (*n* = 80) and services as usual (*n* = 80). Findings indicated that housing stability, substance use, and self-efficacy improved in all conditions. However, 3 months after rental assistance ended, YEH were more likely to be in their own apartment paying rent in housing and supportive services (85%) and housing only (81%) than in service as usual (42%). Of interest is that more YEH in the housing and supportive services condition reported reduced substance use and improved self-efficacy compared to service as usual and housing only. Furthermore, more YEH in housing only reported moderate increasing substance use and no self-efficacy improvement compared to service as usual, suggesting that service as usual outperformed housing only in this regard. In conclusion, housing only appeared to result in less favorable outcomes than service as usual for YEH who also struggled with substance use, mental health and activities of daily living while housing outcomes were best for the two housing arms of the study [25].

An ongoing trial provides evidence for the preventive effects of RRH for non-parenting YEH, 18–24 years, on opioid/other substance use and other concomitant mental health outcomes [24, 33, 60]. Unlike many of the current adult trials, eligible youth do not need to meet criteria for a mental health or substance use disorder to be eligible for the study. In this study, housing and supportive services includes application fees, damage deposit, and furniture and it provides 6 months of rental/utilities assistance rather than 3 months. Supportive services include 6 months of Strengths-Based Outreach and Advocacy in which an advocate links youth to community supports and provides two sessions each of Motivational Interviewing [61] and HIV prevention. The intervention was first tested in a small non-randomized pilot [33, 62]. Findings from the pilot showed that (a) youth maintained their housing at 6 months, (b) marijuana, other drug use and drug use consequences (e.g., arrest, job loss) decreased over time and (c) non-family network size and perceived support from drug using friends decreased over time, likely due to disconnection from street-based peers [33]. Qualitative interviews revealed generally positive experiences with youth believing the intervention had led to improvements in their lives [62]. The provision of housing allowed youth to move out of survival mode, which led to their feelings of self-improvement. Connection to an advocate appeared central to youths’ positive experience. Curry et al. [63] noted that youth often identify strict program rules as barriers to engagement, and struggle with understanding and completing necessary forms and requirements to access community and governmental supports. Youth in the RRH trial felt they could easily contact their advocate and ask them for assistance with a range of things, on their own terms. Overall, pairing housing with less formal support services through advocacy appeared to be well received by the youth. Currently our team is completing a randomized clinical trial (N = 240) to test the RRH developed during the pilot for YEH as compared to usual care/supportive services alone [24]. In the RCT, suicide prevention is also added to the supportive services offered in the pilot [60].

The findings of the pilot study [33] are in contrast to interviews with youth 18–25 years old (N = 26) who received supportive housing for an average of 2 years in NYC [18]. In the latter study, youth paid one-third of the rent and lived in congregate housing. In order to receive housing, youth had to be working, in school or receiving benefits. Although the program identified as using a HF philosophy because youth were not required to engage in treatment services or maintain sobriety to remain in the program, several other aspects are not considered HF. These included the implementation of tenancy rules and congregate housing [64]. Housing outcomes were not reported, but youth reported that “the rigidity of staff roles reinforced to the youth that they were living in a rule driven environment that felt both infantilizing and unresponsive to their needs” [, p. 435). This underscores the vital role of emotionally and instrumentally supportive adults and trust building in programs seeking to assist youth in stabilization efforts.

Taken together, the empirical and qualitative evidence leads to the conclusion that housing is not enough for YEH [17, 25, 56, 62]. Youth need more supports than adults including basic living skills, assistance to maintain housing, money management, shopping for food, attaining education, employment and job training to successfully transition to independent adulthood [55, 65, 66]. Interconnections to functioning across life domains and connections to supportive adults must occur if we are to make progress on ending homelessness and concomitant struggles among youth [17, 56]. While evidence suggests the need to integrate behavioral health and supportive services with housing supports for YEH, ideal design features remain unclear. Our work highlights several lessons learned about optimizing the design of housing interventions for YEH that balance their needs, preferences, and outcomes with feasibility of implementing within complex community settings.

## Lessons learned and recommendations

Even as evidence begins to indicate essential components for addressing the needs of youth, implementation of housing interventions for youth is in its infancy. Challenges finding fair market housing for youth with no credit, criminal justice histories, prior evictions, limited education and few employment experiences, as well as significant substance use and mental health struggles can derail efforts in achieving success. Given our experience testing RRH for parenting and non-parenting YEH specifically (as described in the two trials above), we offer lessons learned from a provider and researcher perspective and recommendations that we hope will benefit future researchers seeking to conduct their own evaluations of housing interventions with youth, and service providers who are implementing and scaling up housing interventions in their communities. We focus our recommendations on the “scattered site” model of HF, which does not require that all YEH are housed within the same building, given that this is the model we are testing. As noted earlier, we incorporated several other HF principles in which youth have choice of where and how they live, develop client-centered goals, are not mandated to receive any services, and sobriety is not required to maintain housing. However, the recommendations below likely have implications for any supportive housing model. They were identified through 4 years of team meetings in which all program staff met biweekly for supervision or general team meetings. Themes from those meetings were identified and summarized below with consensus from all team members.

1. Initial rental
  - Youth often have no credit or poor credit, history of evictions, limited identification, and a criminal record. *Recommendation. Negotiations with landlords to a create pool of housing options for youth is essential to reduce time to housing and prevent a series of unsuccessful applications. Providers/implementers should identify landlords and set up these relationships during the early preparation phase. In addition, providers should clearly outline the logistical process of housing youth upon initiation of partnership with landlords and identify the primary motivations of assisting YEH. By sharing the motives of housing programs and framing the partnership as a team effort, landlords may be more inclined to problem-solve with youth. Another option for large organizations may be to lease units through their agency and then sublease the units to youth. This places a greater responsibility on the agency but allows for quicker placement of youth into housing.*
  - Youth can have large unpaid utility bills from prior housing experiences and utility companies may not turn on the power until these large bills are paid. *Recommendation. In order to prevent delays in housing, providers/implementers should budget to pay past utility bills or seek additional or alternative sources of funding to help pay down these bills. Some utility companies offer programs to mitigate the expenses of qualifying tenants. Providers should establish partnerships with these companies prior to implementation to reduce outstanding balances. Also internet access might not be considered a utility but should be offered because it is often needed to finish a degree, search for employment and stay in touch with healthy forms of support.*
  - Housing location and safety concerns can undermine success in remaining housed. Often housing that will be affordable to YEH are in less desirable areas of town that may have higher rates of crime or are located far away from business areas. YEH may feel especially unsafe living in a neighborhood with high rates of crime if they are living alone for the first time. Furthermore, many YEH have post-traumatic stress disorder (PTSD) and may be prone to hypervigilance, which can be exacerbated by living in an area in which they feel unsafe. Alternatively, if youth are placed in highly residential areas that are far from the business/service area of the city, they may become disconnected from their support networks. YEH often do not have transportation and placing them in housing that is far away from their friends and/or typical hang out spots can lead to them feeling isolated. This may result in them abandoning their housing to be closer to their friends or them allowing people to stay in their housing. *Recommendation. It is important for housing programs to find locations that are desirable to YEH and ensure that they will be able to maintain relationships with their support network. When searching for housing, ensure YEH factor neighborhood and transportation into their decision-making.*
  - If the organization providing rental and utility payments has a rigid financial internal structure, payments to landlords and utility companies could be delayed, jeopardizing youths’ housing and landlord relationships. *Recommendation. An internal taskforce within the institution can expedite payments through tracking and resolving administrative delays. On time payments not only ensure that landlords and other service providers are content with the partnership, but also provides youth a sense of security that their bills are being paid.*
  - Preventing lease termination and eviction while renting
    - Although youth sign their own leases, third party payors, such as a housing program, university or other organization, can be blamed for activities of youth or damage to the apartment, raising concerns among some third-party payors regarding legal risk. *Recommendation. Larger institutions with greater legal infrastructure and insurances that can shoulder the risk might be best positioned to implement these types of housing interventions for YEH. Representatives from the institution should review lease documents and, if necessary, develop addendums for inclusion to ensure both the program implementers and the youth are protected.*
    - Many youth require assistance negotiating with landlords to fix appliances, address leaks and spray for pests, etc. Further, conflicts between youth and landlords are common, and landlords often call advocates to help the negotiation process and request funds to pay for damage. Additionally, available units that are not cost-prohibitive for housing programs or youth tend to require more frequent repair work due to tenant turnover, aging materials, or worn appliances. *Recommendation. Training for advocates might include specialized content on helping youth build conflict resolution and negotiation skills. In addition, program staff should set expectations with youth when they begin the program and/or ask landlords to have a strikes policy. Finally, expectations for apartment furnishings including any working appliances, heating and cooling, and hot water tanks should be outlined and shared with landlords prior to forming housing partnerships, with inspections of units ideally occurring prior to move-in to minimize resident disputes.*
    - It is not uncommon for youth to let friends stay in their apartment who are not on the lease, which is usually disallowed by the lease agreement and can lead to eviction. Similarly, youth or their friends may damage the apartment, breaking windows, or putting holes in walls that can also lead to eviction. Multiple complaints by other tenants due to noise, fighting or calls to the police can also lead to eviction. *Recommendation. Programs may want to consider options that allow youth to lease apartments with roommates of their choice. Many young adults live with roommates to help offset the cost of housing. This may also help alleviate some of the issues around lease violations related to letting friends stay with them. Furthermore, youth need to be reminded of the potential consequences of damaging the apartment.*
    - As youth transition out of housing programs, they often elect to stay in the same apartments and assume responsibility for remaining rent payments. This can be an abrupt financial adjustment for youth and, if they are unable to maintain on-time payments, landlords will proceed with eviction procedures. *Recommendation. Programs should have a clearly defined process for transitioning rent and utility payments to tenants. Landlords should be briefed on this timeline, and the process should be shared with the youth well before the end of the housing program. This will allow youth to plan for the financial burden of housing payments and make informed decisions to prevent possible evictions.*
2. Supportive services
  - The research team interviewed youth receiving supportive services in the project finding that youth reported feeling cared about by their advocate was very important to uptake of any offered services [62]. YEH need to believe that the advocate is invested and interested in helping them for them to trust the advocate and accept support services. *Recommendation. It is important that agencies hire staff who are flexible and understand the importance of letting youth guide the amount of support they want to accept. Especially early on, youth may be more responsive to the tone of meetings than their frequency. Agencies need to train staff on strength-based approaches that help staff develop positive rapport with youth.*
  - The focus of the first several weeks of advocacy is on housing, obtaining identification and ensuring basic needs are met (food, safety, medical care, etc.). As the housing crisis stabilizes, youth and advocates focus on other high needs areas including education, employment, money management, mental health and substance use. *Recommendation. Serving priority goals for the youth builds relationship and self-efficacy. Training advocates on how to best connect to these resources will ensure that foundational needs are met as promptly as possible. Developing and distributing a paper handout or digital resource document with available services can provide youth with immediate access to information while reinforcing youth’s problem-solving abilities.*
  - Youth vary in their preferences of visit frequency. Some youth request consistent meetings with advocates over the 6-month advocacy period, while other youth are slow to build trust and request fewer meetings, usually focused on instrumental assistance. In general, the frequency of meetings is higher at the beginning of the advocacy relationship, with meetings of up to 2, 3 times/week. After 2–3 months, frequency tapers to one time/week or every other week as youth stabilize. *Recommendation. Allowing an “any door is the right door” policy will provide the greatest flexibility. Advocates should feel empowered to discuss visit preferences with youth early on, which will be helpful information for scheduling purposes and will also reinforce to youth that their needs are valued.*
  - Youth often no-show appointments with advocates usually due to the chaos and unpredictability inherent in their lives. Organizations might find it difficult to implement and sustain these interventions if they are funded using a fee-for-service model that only reimburses providers for completed service visits. *Recommendation. Avoid advocate fee for service models: alternative financing arrangements will be important to explore. Advocates should also practice patience with missed appointments and adjusted plans. By modeling clear communication and boundaries to youth, advocates can demonstrate reasonable accountability and minimize negative communications.*
  - Youth may disappear for weeks at a time and reappear. Methods of communication can be unreliable as phones may be lost, cell services may be disconnected, and social media accounts may be locked. Youth might travel out of state, be sent to jail, be hospitalized and/or avoid the advocate for various reasons such as relapse. Unfortunately, some youth may also die due to violence, overdose, or suicide. *Recommendation. Advocates may need to adjust expectations for meeting consistency and be prepared to pick up where they left off, weeks prior, with youth. Advocates may also plan ahead by discussing alternative contact methods and ways to reconnect with youth. Locator documents with alternative contacts should be confirmed with youth regularly. In addition to already established methods of contacting youth, advocates should be trained on tracking practices to locate youth through social media and inmate listings.*
  Some of the youth experiences above have direct implications for training advocates. In particular, training advocates to sequence intervention components, and to understand and calibrate meeting frequency to youth preferences, as well as forming realistic expectations of youth, will be essential to success.
3. Advocate experiences
  - Frustration coordinating with landlords can be high as advocates seek to preserve the rights of youth. *Recommendation. Supervisors should help advocates develop realistic expectations and continuously support advocate efforts. Additionally, by outlining housing program processes and expectations to landlords at the outset of partnerships, landlord and advocate expectations may be more easily reconciled.*
  - Secondary trauma can occur. Most youth experience violence and victimization, suicidal thoughts and attempts. High levels of advocate empathy combined with feelings of powerlessness can lead to a sense of learned helplessness. Prior unresolved traumas among advocates can be triggered by youths’ experiences. *Recommendation. Supervisors need to monitor advocate mental health and make appropriate referrals when necessary. Fostering a team-orientated environment where advocates and supervisors communicate and collaborate regularly will allow for supervisors to adequately monitor advocates and for advocates to feel supported when these feelings arise.*
  - Advocates may experience direct or secondary racism, bias, or discrimination when working with service providers related to their assigned youth. These experiences may include poor or differential treatment, invisibility, or denial of reasonable accommodations. Furthermore, the advocate may be more likely to identify these acts of racism, bias, and discrimination due to less frequent encounters with these experiences than YEH. *Recommendation. The advocate can use these encounters to model to the youth how to process and navigate future negative interactions. Furthermore, advocates can work with their supervisor to address their own experiences and develop a response strategy when experiences of injustice, racism and system bias occur.*
  - Youths’ needs do not fit into a 9 to 5 workday. Advocates are on-call for youth in off-hours, which can lead to burn-out and unresponsiveness to youth. Also, advocates’ hours may vary from week to week depending on the current phase of intervention for their clients. *Recommendation. To the extent possible, implementers should balance caseloads based on client need. Also, providers in the homeless services field are often underpaid compared to other disciplines. It is important for agencies to fairly compensate advocates for the extent of their work. Advocates should also work together to assist with time-sensitive tasks if the YEH’s assigned advocate is unavailable. For example, if a client needs to tour an apartment and the assigned advocate is unavailable.*
  - Advocates might observe quick change among youth. The advocate-youth relationship can reinforce the importance and power of the human bond in affecting positive change. For some youth, this relationship is the first experience of unconditional positive regard. *Recommendation. Reinforce to the advocates the power of the advocate-youth bond. In addition to resource connections for basic needs, advocates should prioritize establishing a genuine relationship with the youth during the early phase of advocacy. Throughout the program, advocates should reinforce the unconditional nature of their concern for the youth.*
  - Multiple advocacy experiences, such as those described above, have implications for advocate burnout and turnover. *Recommendation. A strong and supportive work climate and supervision are important for high quality implementation, service delivery, and sustainment. Supporting the mental health of advocates through providing referrals and a safe environment to discuss experiences with youth may be essential for preventing secondary trauma experiences and burnout. It is also important to help advocates develop realistic boundaries to prevent them from overextending themselves.*
  - Advocacy can be a difficult position. Not all individuals will have the temperament or skills needed to provide this service to youth. *Recommendation. The advocate’s relationship with their client often has a powerful effect on the youth, so it is important that advocates be well trained in advance and supported throughout their work. For implementation consistency and reliability, programs should also be responsive to youths’ perceptions of advocacy services by establishing a survey process completed by a non-advocate provider.*

## Conclusion

Efforts to end homelessness have turned from a treatment first model to a HF model as the gold standard [44]. However, few randomized trials supporting the effectiveness of housing programs using a HF philosophy have been completed. Several of those trials to date do not adhere to HF principles, are not randomized, and do not include youth—limiting our understanding of the effectiveness of housing using HF principles for YEH. However, a growing evidence base suggests that housing alone is insufficient for youth experiencing homelessness [17, 25, 56]. YEH are in the midst of important developmental milestones, and these processes often co-occur with critical losses of important social and institutional supports as well as intrapersonal struggles such as substance use and mental health. Therefore, intervention supports for youth should differ from those of older adults. For example, youth need assistance with activities of daily living and developmental supports associated with identity development—including racial, sexual and gender identity development, as well as linkages to supportive networks and services to address prior trauma and ongoing substance use and mental health struggles.

Currently, our team has completed the largest randomized trial of RRH using HF principles with YEH (N = 240) to date which focused on parenting youth [25] and are conducting a second HF trial with non-parenting YEH (N = 240) [24, 33, 60]. Several hard lessons have been learned regarding implementing RRH, service provision, as well as the psychological effects on advocates associated with intervening on youth’s behalf. We offered several recommendations to address these challenges in an effort to assist researchers and providers seeking to end homelessness among youth.

## Data Availability

The data generated and reported in the current study is not publicly available.
